# Coexistence of three sympatric cormorants (*Phalacrocorax* spp.); partitioning of time as an ecological resource

**DOI:** 10.1098/rsos.160175

**Published:** 2016-05-18

**Authors:** Mylswamy Mahendiran

**Affiliations:** Department of Environmental Studies, University of Delhi, New Delhi 110007, India

**Keywords:** behaviour, cormorants, diving time, resource partition, sympatric, temporal

## Abstract

Resource partitioning is well known along food and habitat for reducing competition among sympatric species, yet a study on temporal partitioning as a viable basis for reducing resource competition is not empirically investigated. Here, I attempt to identify the mechanism of temporal partitioning by intra- and interspecific diving analyses of three sympatric cormorant species at different freshwater wetlands around the Delhi region. Diving results indicated that cormorants opted for a shallow diving; consequently, they did not face any physiological stress. Moreover, diving durations were linked with seasons, foraging time and foraging habitats. Intraspecific comparison suggested that cormorants spent a longer time underwater in early hours of the day. Therefore, time spent for dive was higher in the forenoon than late afternoon, and the interspecific analysis also yielded a similar result. When *Phalacrocorax niger* and *Phalacrocorax fuscicollis* shared the same foraging habitat, they tended to differ in their foraging time (forenoon/afternoon). However, when *P. niger* and *Phalacrocorax carbo* shared the same foraging time, they tended to use different foraging habitats (lentic/lotic) leading to a mechanism of resource partitioning. Thus, sympatric cormorants effectively use time as a resource to exploit the food resources and successful coexistence.

## 1. Background

In heterogeneous environments, niche differentiation functions as a mechanism of coexistence among competitors [1], usually along habitats, food resources, time axis or a combination of them. Niche differentiation facilitates ecological diversification through coexistence by avoidance of direct confrontation (interference competition) or reduction of resource overlap (resource competition) [2]. Sympatric species evolve different activity patterns to reduce interspecific resource and interference competition [1,3–5]. Temporal partitioning is a viable mechanism for reducing resource competition and sometimes trade-offs in foraging ecology as well [6–8]. However, time appeared to be a farless common mode of resource partition among sympatric species when compared with habitat [9] and food type [3,9]. Although ecological interactions over time have been observed in a number of communities *viz*., reptiles [10,11], birds [12] and mammals [6], the mechanism of time as an ecological resource is still poorly understood [3,12,13].

It has been well documented that birds could identify the subtle differences in the prey environment [14], even within prey species; for example, they are able to distinguish prey characteristic features [15–18] for effective use of resources. In freshwater wetlands of the Delhi region, three sympatric cormorant species *viz*., little cormorant (*Phalacrocorax niger*) (hereafter *P. niger*), Indian cormorant (*Phalacrocorax fuscicollis*) (hereafter *P. fuscicollis*) and large cormorant (*Phalacrocorax carbo*) (hereafter *P. carbo*) [19–21] forage together which are ideal models for a comparative study of diving behaviour and flexibility in foraging choices [2,22]. Foraging consists of dive time (hereafter = *T*_D_) and surface/pause time (hereafter = *T*_P_), and each dive follows a period on the surface for respiration [23,24]. It is well established that water depth [16,25], age [26], sex [27], underwater predation pressure [28] and kleptoparasitism [16] influences diving performance. However, intra- and interspecific species interactions along with environmental factors that affect diving behaviour receive little attention [5,11].

Here, I assess the diving behaviour of three cormorant species at different freshwater wetlands in the Delhi region, to identify interactions with environmental factors *viz*., breeding seasons, foraging time and foraging habitats, to explore adjustments that they make to use resource effectively for successful coexistence.

## 2. Material and methods

### 2.1. Study area

**Figure 1.**
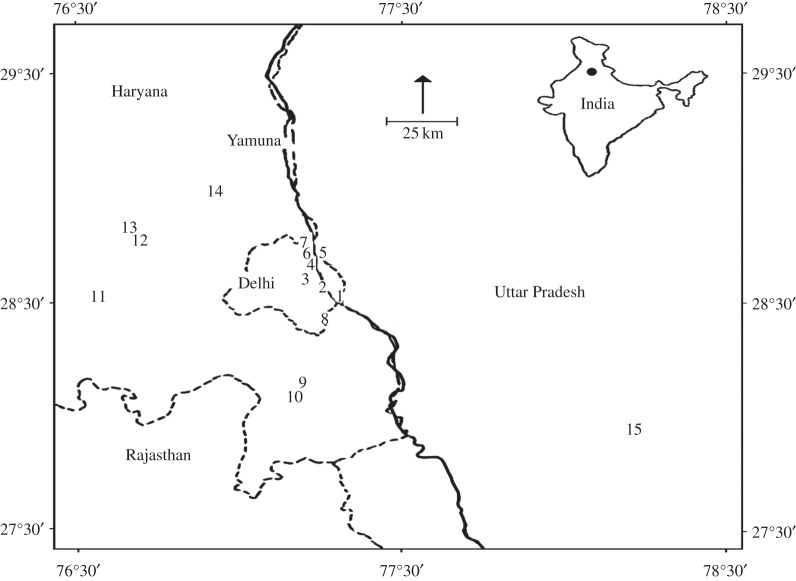
Numbers indicate the location of 15 freshwater wetlands and their corresponding names are: (1) Okhla Barrage Bird Sanctuary, (2) Indraprastha Thermal Power Station Pond, (3) Old Fort Lake, (4) Wazirabad Barrage, (5) Sonia Vihar, (6) Yamuna Biodiversity Park, (7) Jagatpur, (8) Bhatkal Lake, (9) Mandkaula, (10) Khanoli, (11) Bhindawas Bird Sanctuary, (12) Kharhar, (13) Sampla, (14) Mohamedabad and (15) Sheikha Jheel. State boundaries are shown by dotted lines. Inset shows the location of the study area (filled circle) in India.

Field studies were conducted from January 2004 to December 2010 at different freshwater wetlands scattered within a radius of 180 km around Delhi (figure 1). Broadly, the area falls under the semi-arid Punjab plains 4A and upper Gangetic plains 7A of the biogeographical classification [29]. Wetlands were classified as lentic or lotic, and both the habitats were located inside protected areas (sanctuaries and parks) as well as in non-protected areas. Lotic wetlands *viz*., Okhla Barrage Bird Sanctuary, Wazirabad Barrage and Sonia Vihar wetlands were connected directly to the river Yamuna [22].

### 2.2. Diving observations

Cormorants were observed with binoculars (7′ × 50′′) and a telescope (15×). Focal animal sampling [30] was adopted, and observations were made from land at a distance of approximately 25–300 m as prescribed [31]. Each foraging bout was estimated from the time an individual started foraging until it finished completely, and a typical post-feeding behaviour [32] was used to ascertain the completion of foraging. A foraging bout was regarded as disturbed when a focal bird abruptly terminated foraging due to a disturbance event, caused by human activity or some other agent. Care was taken not to lose track of the focal bird. When there was doubt about the identity of a focal bird, especially, when two foraging birds crossed paths, or if a bird disappeared permanently from the view, or if the diving bout sequence was less than five dives, then that record was summarily discarded. Only serious dives were taken for analysis, and very short dives, sometimes little more than head dips were omitted for analysis as it was considered to be commuting dives [25]. A typical dive cycle [24,33,34] comprised: (i) pause time during which a bird replenishes oxygen at surface, (ii) descending time—a bird moves from water surface to foraging area, (iii) foraging time—a bird spends for foraging at the bottom, and (iv) ascending time—a bird returns to surface. Observations on *T*_P_ and *T*_D_ were collected, and *T*_D_ represented the union of descending, foraging and ascending time. The time for which a focal bird remained on surface *T*_P_ and underwater *T*_D_ was estimated to the nearest second by using a digital electronic stopwatch. Observations were made on relatively calm days, and field data collection was avoided on rainy or heavy windy days. Observations were not altered on any account, for any other reasons [25].

Time, date, wetland name, focal species details and notes of the weather were noted on data recording sheets. Months of July–September, October–November and December–February were considered as breeding seasons for *P. niger, P. fuscicollis* and *P. carbo*, respectively [19,20]. Therefore, the diving behaviour observed within those months was categorized as breeding dives, and that in the remaining months was considered as non-breeding dives for each species, accordingly. Although seasonal differences of diving time were an intrinsic biological character, for the convenience of analysis, it was included along with other extrinsic environmental parameters. Further, dives were segregated as lentic or lotic depending on the nature of wetland in which those observations were made. Wetlands of Okhla Barrage Bird Sanctuary, Wazirabad Barrage and Sonia Vihar were classified as lotic wetlands, and the remaining as lentic wetlands (figure 1).

### 2.3. Data analysis

The data were entered into a worksheet, and analysis was performed using Minitab 17.1.0. A Kolmogorov–Smirnov normality test was employed to check the normality of the data. GLM-ANOVA was employed to test the differences with respect to *T*_D_, *T*_P_ and bout length among the three species of cormorants. To establish a relationship between *T*_D_ and *T*_P_ for each species separately, linear regressions were employed in which *T*_P_ was made a dependent variable and *T*_D_ as an independent variable, and limitations pertaining to regression were checked [18]. The dives within bouts are mutually related in a way that bouts from different dive cycles are not; for example, within a species, smaller or younger birds would typically dive for shorter times. Therefore, this non-independence of bouts was taken into account in the analysis following the method of Lea *et al.* [35]. To test the dive of cormorants as either reactive or anticipatory, mean *T*_P_ for the bout of dives concerned was first subtracted from each *T*_P_, to give what can be called ‘residual pause times’ [35]. *T*_D_ values were then submitted to multiple regression using dive bouts, residual preceding pause time (*T*_P_-Pre) and succeeding pause time (*T*_P_-Suc).

The intraspecific variability of *T*_D_ was tested in relation to environmental variables as described later. The coefficient of variation (CV) of *T*_D_ and *T*_P_ was high; therefore, the data were subjected to further scrutiny among the species to explore the influence of other environmental factors. Both intraspecific and interspecific analyses were performed. For intraspecific analysis, GLM-ANOVA and *post hoc* tests were done. As the dependent variables were categorical representing species, nominal logistic regression models were employed to detect the interspecific interactions and influence of environmental variables. Therefore, the dependent categorical values of species (coded as: *P. niger*: 0; *P. fuscicollis*: 1; *P. carbo*: 2) regressed upon the environmental variables *viz*., foraging habitat (lentic/lotic), foraging time (forenoon: before 12.00/afternoon: after 12.00) and the seasons (breeding/non-breeding) of the three sympatric species of cormorants. All the results are expressed as mean ± s.d.

## 3. Results

Diving observations of 1012 dive cycles which comprised 63 diving bouts from different individuals of three cormorant species were collected from 15 freshwater wetlands in the Delhi region (table 1).

**Table 1.** Variations (mean ± s.d.) in the diving behaviour of three species of cormorants, their sample sizes are given in parenthesis (n.s., non-significant; CV, coefficient of variation).

parameters	*P. niger*	*P. fuscicollis*	*P. carbo*
dive time (s)^a^	14.72 ± 6.65 (538)	19.82 ± 8.54 (204)	18.26 ± 7.13 (270)
dive time CV (%)	45	43	39
pause time (s; n.s.)	6.36 ± 3.82 (538)	6.31 ± 3.2 (204)	6.09 ± 2.78 (270)
pause time CV (%)	60	51	46
dives per bout (n.s.)	16.3 ± 8.57 (33)	15.69 ± 7.62 (13)	15.89 ± 3.81 (17)
length of the bout (s; n.s.)	356.1 ± 204.3 (33)	410.2 ± 168.2 (13)	386.7 ± 113.5 (17)
*T*_D_/*T*_P_	2.74	3.52	3.28

a GLM-ANOVA, *p *< 0.001.

### 3.1. Variations in *T*_D_ and *T*_P_

*Phalacrocorax niger*, smallest in terms of body-size, had the shortest mean *T*_D_ among three species (14.72 ± 6.65; *n* = 538). Significant differences in *T*_D_ (GLM-ANOVA, *F*_2,1009_ = 45.951; *p* < 0.001) were observed when compared among three species of cormorants (table 1). As per the *post hoc* Scheffe test, *T*_D_ of *P. niger* significantly differed from that of both *P. fuscicollis* (*p* < 0.001) and *P. carbo* (*p* < 0.001). However, the difference between *P. fuscicollis* and *P. carbo* was not significant (*p* < 0.065).

**Figure 2.**
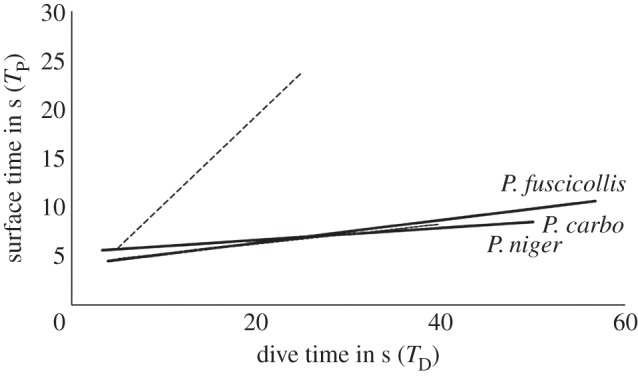
Represents the types of diving adaptations observed in cormorant species. The ordinates represent the amount of time the cormorant remains underwater during diving, denoted as (*T*_D_), and the time it spends on the surface to replenish air for breathing immediately after the dive, denoted as (*T*_P_). The dotted line represents diving behaviour of marine cormorants [26,36]. The other three straight lines represent the linear regression fit-line using dive time (raw data) of cormorants.

Further, *T*_P_ was non-significant (n.s.) among three species of cormorants (GLM-ANOVA, *F*_2,1009_ = 6.47; *p* < 0.580, n.s.), and estimated as 6 s in all three species (table 1) which indicates their role of respiration and physiological limits. When *T*_P_ was regressed upon *T*_D_, values of the slopes were extremely low for all three species suggesting that these species opted for shallow diving (figure 2). The regression equations of three species were estimated as follows: *P. niger*: *T*_P _= 0.0926 *T*_D_ + 4.9909 (*r*^2^ = 0.0258, *p* < 0.001), *P. fuscicollis*: *T*_P _= 0.116 *T*_D_ + 4.015 (*r*^2^ = 0.0959*, p* < 0.001) and *P. carbo*: *T*_P _= 0.0997 *T*_D_ + 4.2702 (*r*^2^ = 0.0653, *p* < 0.001) (figure 2). Although *T*_D_ includes various activities *viz*., ascending, searching for prey, vigilance from predator and descending, CV of *T*_D_ was lower than that of *T*_P_ (range 45–60%), mainly due to the handling time of prey plus respiration at the surface. However, there was a significant positive relationship between *T*_D_ and *T*_P_ in all the three species of cormorants after controlling for the effect of non-independence of diving bouts (table 2). Further, dives were reactive in nature in all the three species of cormorants. In *P. fuscicollis*, quite a substantial difference was noted, which happened to fall short of significance (*p* < 0.068) in favour of anticipatory breathing (table 2).

**Table 2.** Multiple regression analysis estimating the relationship of dependent variable *T*_D_ with *T*_P_ after controlling for the effect of difference in dive bouts. (Succeeding pause time (*T*_P_-Suc) indicates reactive dive; preceding pause time (*T*_P_-Pre) indicates anticipatory dive.)

				*t*-test		ANOVA			
species	set	predictors	coefficient ± s.e.	*t*-value	*p*-value	model *R*^2^ (%)	d.f.	*F*-value	*p*-value
*P. niger*									
	1	*T*_P_	0.368 ± 0.0647	5.68	0.001	40.7	1, 504	32.25	0.001
	2	*T*_P_-Suc	0.368 ± 0.0647	5.69	0.001	40.8	1, 504	32.34	0.001
	3	*T*_P_-Pre	−0.001 ± 0.0723	−0.01	0.985	36.2	1, 468	0.000	0.985
*P. fuscicollis*									
	1	*T*_P_	0.250 ± 0.162	1.54	0.126	42.0	1, 190	2.360	0.126
	2	*T*_P_-Suc	0.255 ± 0.163	1.57	0.118	42.0	1, 190	2.460	0.118
	3	*T*_P_-Pre	0.396 ± 0.216	1.83	0.068	42.4	1, 178	3.360	0.068
*P. carbo*									
	1	*T*_P_	0.569 ± 0.135	4.21	0.001	39.4	1, 252	17.74	0.001
	2	*T*_P_-Suc	0.566 ± 0.135	4.19	0.001	35.3	1, 252	17.57	0.001
	3	*T*_P_-Pre	−0.029 ± 0.146	−0.20	0.844	35.9	1, 235	0.400	0.844

The *T*_D_ and *T*_P_ ratio of *P. niger* were lowest among the three species. As expected, a positive gradient along body-size with a ratio of 2 : 1 for *P. niger* and 3 : 1 for *P. carbo* was estimated (table 1). Although buoyancy force depends on body-size, it did not make any difference in mean foraging bout length (GLM-ANOVA, *F*_2,62_ = 0.480; *p* < 0.621, n.s.). Much of the differences in diving activity followed a pattern along body-size gradation representing their physiological limits.

### 3.2. Intraspecific diving interactions

Intraspecific variations of *T*_D_ values were examined in relation to selected environmental variables *viz*., foraging habitat, time of the day and breeding seasons. For each species, possible effects of confounding variables were removed by using only a subset of the data. For instance, testing if the *T*_D_ values differ between lentic and lotic habitats, only data of Okhla Barrage Bird Sanctuary and Jagatpur were used because they were close to each other. It was ensured that selected datasets were as close as possible, in terms of the time of the day as well as the month, so that the effects of these variables do not confound the analysis. Except for time of the day, results do not conclusively show that *T*_D_ values differed in relation to environmental variables taken. In several datasets of *P. niger*, *P. fuscicollis* and *P. carbo*, mean *T*_D_ values of the time slot prior to 11.00 were higher than late afternoon or evening (table 3), indicating that birds spent longer time underwater in early hours of the day.

**Table 3.** Intraspecific variations (*T*_D_) of cormorants and corresponding dive time under different time slots. (Refer to figure 1 for the details of site numbers, and values in parenthesis are sample sizes. M-dash denotes insufficient data.)

		mean dive time under different time slots					
species	site no	I (less than 11.00)	II (11.00–14.00)	III (14.00–16.00)	IV (greater than 16.00)	GLM-ANOVA	*post hoc* Scheffe test
*P. niger*	7	16.1 ± 6.3 (11)	7.4 ± 1.7 (7)	9.2 ± 3.3 (93)	11.8 ± 5.03 (9)	*F = 13.141*	*I* versus II**
						*p = 0.001*	versus III**
*P. fuscicollis*	1, 2, 11	17.8 ± 7.01 (23)	23.8 ± 5.20 (17)	20.1 ± 7.04 (33)	—	*F = 3.985*	*I* versus II*
						*p = 0.02*	
*P. carbo*	7, 9, 10, 12	22.8 ± 6.8 (49)	10.6 ± 2.05 (18)	13.9 ± 5.3 (42)	18.3 ± 10.3 (13)	*F = 22.876*	*I* versus II**
						*p = 0.001*	versus III**

*p < 0.05; **p < 0.001.

### 3.3. Interspecific diving interactions

The environmental variables *viz*., breeding seasons, habitats and foraging time, had an independent effect on *T*_D_; micro- and macro-level effects were identified among three cormorant species.

At macro-level, *T*_D_ varied significantly between breeding and non-breeding seasons (*P. niger* and *P. fuscicollis* (*p* < 0.001); *P. niger* and *P. carbo* (*p* < 0.001). However, no such variations were noted in *T*_P_ of cormorants (table 4). At the micro-level, a clear segregation and interactions over habitat selection (lentic/lotic) and foraging time (forenoon/afternoon) were recorded.

**Table 4.** A nominal logistic regression model to explore the influence of intrinsic and extrinsic environmental factors on the diving behaviour of sympatric cormorants. (Log-likelihood = −898.990; test that all slopes are zero: *G* = 248.783, d.f. = 10, *p*-value = 0.001, dependent variables are species, coded as 0: *P. niger*; 1: *P. fuscicollis*; 2: *P. carbo*.)

	logit (1)			logit (2)		
	*P. fuscicollis*/*P. niger*			*P. carbo*/*P. niger*		
predictors	co-efficient	s.d.	*p*-value	co-efficient	s.d.	*p*-value
constant	−1.275	0.338	0.001	−1.682	0.331	0.001
breeding seasons (breeding 1, non-breeding 2)	−0.689	0.212	0.001	0.712	0.168	0.001
*T*_D_ (dive time)	0.092	0.124	0.000	0.058	0.012	0.001
*T*_P_ (pause time)	−0.019	0.028	0.483	−0.039	0.028	0.159
foraging time (forenoon-1, afternoon-2)	−0.497	0.112	0.001	−0.139	0.103	0.177
habitats (lentic-1, lotic-2)	0.586	0.302	0.052	1.861	0.235	0.001

A significant diurnal variation in foraging time (forenoon/afternoon) was observed between *P. niger* and *P. fuscicollis* (*p* < 0.001); subsequently, no significant difference was noted in the selection of foraging habitats (*p* < 0.052) (table 4). In other words, the chance of a sighting of both species (*P. niger* and *P. fuscicollis*) at the same site was possible; however, it differed in their foraging time between forenoon and afternoon. Interestingly, the results were exactly opposite when the comparison was made between *P. carbo* and *P. niger*. No significant difference in foraging time was observed between them (*p *< 0.117). However, a significant difference in the selection of foraging habitats (*p* < 0.001) was observed (table 4). Both species foraged at the same time, but differed in their foraging habitats. When *P. carbo* selected lotic habitats, the smaller *P. niger* restricted to lentic water-bodies (table 4).

## 4. Discussion

### 4.1. Diving behaviour

Diving behaviour of cormorants combines the key physiological and ecological factors [24]. *T*_P_ explains the role of respiratory physiology (ability to obtain oxygen at the surface) and *T*_D_ explains ecological factors that affect the diving behaviour. Anaerobic dive was reported in deeper wetlands, and cormorants were recorded to dive to great depths of approximately 99 m [36,37] in marine habitats. As the wetlands in the study areas are shallow (less than 10 m) and monsoonal in nature [22], birds would prefer energetically profitable dives. Further, *T*_P_ was more or less uniform of 6 s in all the species (figure 2); therefore, diving results support the predictions of optimal breathing models [33–35] and aerobic diving. If animals operate within their oxygen capacity, then I would not expect any correlation between *T*_D_ and *T*_P_ [35].

An attempt was made to look for a point of inflection in the *T*_D_ and *T*_P_ regression curve, as in other marine studies, [26,36] to check whether birds crossed the aerobic diving limit. No such inflection was noticed (figure 2), suggesting that cormorant species did not perform any deeper dives leading to physiological stress. If the cormorants had incurred a diving cost (say oxygen debt), then it would have reflected in higher slope values of *T*_D_/*T*_P_ regressions. It re-confirmed that *T*_P_ was not proportionally longer with *T*_D_, and cormorants were not undergoing any oxygen depletion.

Here, only a small spectrum of diving ability of cormorants was accounted, and if the habitat was deep enough, then *T*_D_ would have extended a longer time in underwater. It was noted that air-breathing divers changed their dive tactics, time allocation depending on the aim of the dive and surfaced without depleting their estimated stores of oxygen [11]. The results of this study showed enough empirical support with earlier ideas [24,35] *viz*., dives are not always terminated solely on the basis of oxygen or finding an acceptable prey or close to aerobic diving limit or perhaps beyond. The dives were reactive in all three species of cormorants, which clearly supports the assumption that *T*_P_ should depend on the preceding dive time, satisfying the condition of reactive breathing. Although the results were not in favour of the anticipatory breathing mode, where dive time depends on the preceding surface time, in any of the three cormorants, a slight edge of anticipatory dive was observed in *P. fuscicollis* (table 2).

### 4.2. Time as ecological resource

It is well documented in birds that they tend to make finer adjustments to use resources, distinguish subtle differences in the foraging environment [14], compensate for foraging time [7], and distinguish prey dimensions [8,15,16] and colour [17]. Therefore, the adjustments that the bird makes for effective use of resources, which leads to coexistence, would be clearly understood through intra- and interspecific analysis [2,5]. Resource partitioning of cormorants with respect to food [9,16] and habitat [23,36,38,39] is known, and each cormorant species have their unique diving time [40] that differed significantly from each other [38]. Diving activity followed a pattern along body-size gradation representing their own typical physiological limits [2,16,35,41]. However, cormorants’ diving efficiency (*T*_D_ and *T*_P_ ratio) was high, indicating interspecific interactions and the influence of environmental factors [5,11,38] that affected the diving behaviour, other than the physiological differences.

Intraspecific analysis of *T*_D_ suggested that foraging differences were noticed, in the case of *P. niger* and *P. carbo*, over the time of day. However, no satisfactory conclusion was found on habitats and seasons owing to the lack of confounding sub-datasets. Interspecific interactions over the season (macro-level) and the time of day (micro-level) influenced the diving behaviour of cormorants. The difference in *T*_D_ between breeding and non-breeding was obvious as birds need to find food for themselves and their nestlings. Therefore, it is expected that birds would undertake an extra foraging effort during breeding time and sexual difference in foraging also possible [27].

At micro-level, the diving difference was observed with extrinsic environmental variables *viz*., foraging habitat (lentic/lotic) and foraging time of the day (forenoon/afternoon). Changes in the diving behaviour reflect differences in prey abundance, prey behaviours in foraging habitats [42] and predation pressure [28]. Habitats are known to exert a strong influence on the temporal distribution of prey, which ultimately influenced the distribution pattern of cormorants [43]. When *P. niger* and *P. fuscicollis* selected the same habitats, a segregation in foraging time was observed; however, the result was reverse in *P. niger* and *P. carbo*, which indicated a resource partitioning to minimize competition.

The probable ecological factors that drive this apparent time sharing, perhaps, could be the prevailing differences in the diurnal cycles of prey species, and preference of prey size selection among cormorants. It was experimentally proved that cormorants caught fishes that ranged from 30 to 140 mm, and the prey sizes taken by each species were significantly different [16]. Further, a significant difference in handling time was noted for the same size of fishes, which indirectly indicated a preferential selection of different prey species [22]. Unlike the European shag (*Phalacrocorax aristotelis*), all cormorant species typically brought fish to the water surface, one at a time [21], so it attracts issues of kleptoparasitism as well [16]. It was noted that there was a slight variation in foraging time between winter and summer with the cormorants being visual pursuit divers; so the influence of day light was obvious [22]. These factors, perhaps, either on their own or in combination of others, mediate the apparent time-sharing among cormorant species. Thereby, the three species of cormorants successfully coexist through partitioning of time to exploit the resources effectively.

## Data accessibility

The supporting data are available on Dryad: http://dx.doi.org/10.5061/dryad.93k26.
